# GSDMD-dependent pyroptotic induction by a multivalent CXCR4-targeted nanotoxin blocks colorectal cancer metastases

**DOI:** 10.1080/10717544.2022.2069302

**Published:** 2022-05-09

**Authors:** Rita Sala, Elisa Rioja-Blanco, Naroa Serna, Laura Sánchez-García, Patricia Álamo, Lorena Alba-Castellón, Isolda Casanova, Antonio López-Pousa, Ugutz Unzueta, María Virtudes Céspedes, Esther Vázquez, Antonio Villaverde, Ramon Mangues

**Affiliations:** a Biomedical Research Institute Sant Pau (IIB-Sant Pau), Barcelona, Spain; b CIBER en Bioingeniería, Biomateriales Y Nanomedicina (CIBER-BBN), Madrid, Spain; c Josep Carreras Research Institute, Barcelona, Spain; d Institut de Biotecnologia I de Biomedicina, Universitat Autònoma de Barcelona, Bellaterra, Spain; e Departament de Genètica I de Microbiologia, Universitat Autònoma de Barcelona, Bellaterra, Spain; f Department of Medical Oncology, Hospital de la Santa Creu I Sant Pau, Barcelon, Spain

**Keywords:** Metastasis, CXCR4, colorectal cancer, targeted nanoparticle, PE24 exotoxin, GSDMD, pyroptosis

## Abstract

Colorectal cancer (CRC) remains the third cause of cancer-related mortality in Western countries, metastases are the main cause of death. CRC treatment remains limited by systemic toxicity and chemotherapy resistance. Therefore, nanoparticle-mediated delivery of cytotoxic agents selectively to cancer cells represents an efficient strategy to increase the therapeutic index and overcome drug resistance. We have developed the T22-PE24-H6 therapeutic protein-only nanoparticle that incorporates the exotoxin A from *Pseudomonas aeruginosa* to selectively target CRC cells because of its multivalent ligand display that triggers a high selectivity interaction with the CXCR4 receptor overexpressed on the surface of CRC stem cells. We here observed a CXCR4-dependent cytotoxic effect for T22-PE24-H6, which was not mediated by apoptosis, but instead capable of inducing a time-dependent and sequential activation of pyroptotic markers in CRC cells *in vitro*. Next, we demonstrated that repeated doses of T22-PE24-H6 inhibit tumor growth in a subcutaneous CXCR4^+^ CRC model, also through pyroptotic activation. Most importantly, this nanoparticle also blocked the development of lymphatic and hematogenous metastases, in a highly aggressive CXCR4^+^ SW1417 orthotopic CRC model, in the absence of systemic toxicity. This targeted drug delivery approach supports for the first time the clinical relevance of inducing GSDMD-dependent pyroptosis, a cell death mechanism alternative to apoptosis, in CRC models, leading to the selective elimination of CXCR4^+^ cancer stem cells, which are associated with resistance, metastases and anti-apoptotic upregulation.

## 1. Introduction

Metastatic dissemination remains the main cause of death in colorectal cancer (CRC) as in other tumor types. Despite advances in systemic therapy, the five-year survival rate of metastatic CRC patients is only 14% (Crooke et al., 2018), being the acquisition of resistance to therapy the major reason for treatment failure. Nearly half of metastatic CRC patients are resistant to chemotherapy regimens, which are mainly based on 5-fluorouracil (5-FU) and oxaliplatin (Virag et al., 2013; Francipane et al., 2019). Cancer cells can display intrinsic resistance or acquired resistance developed along with treatment, which involves agent-specific mechanisms that widely vary among genotoxic agents and molecularly targeted drugs (Pan et al., 2016). Thus, cancer cells activate different mechanisms to escape from therapy-induced death, and apoptosis evasion, as a result of genetic and epigenetic alterations, one of the most prevalent (Hanahan & Weinberg, 2011). Accordingly, the most frequent alterations found in CRC, and also in other solid tumors, involve proteins of the Bcl-2 family, such as upregulation of Bcl-xL or Mcl-1 anti-apoptotic proteins (Schulze-Bergkamen et al., 2008; Song et al., 2020). Therefore, new anticancer therapies that trigger mechanisms of action alternative to apoptosis induction, could overcome drug resistance mediated by apoptosis evasion, expecting to have a significant impact on cure rates and patient survival.

Furthermore, targeted drug delivery aims at increasing the effectiveness of anticancer agents while reducing systemic toxicity (Singh & Lillard, 2009). Here, we developed self-assembling protein-based nanoparticles with intrinsic cytotoxic activity, to explore their targeting selectivity and the possible activation of cell death pathways alternative to apoptosis. For that purpose, we chose to study the anticancer effect of a novel protein-only nanoparticle that actively targets the CXCR4 receptor, whose overexpression has been observed in many tumor types and correlates withpoor survival, dissemination, and recurrence in CRC patients (Kim et al., 2005) and accommodates the de-immunized 24 kDa catalytic domain of the *Pseudomonas aeruginosa* exotoxin A (PE24). This exotoxin is in the same position as the GFP protein in the T22-GFP-H6 nanocarrier (Falgàs et al., 2020), forming self-assembled nanoparticles that incorporate several monomers per nanotoxin (estimated to be 10–12 in a similar construct) (Rueda et al., 2015).

Interestingly, we previously reported the capacity of a different nanotoxin that incorporates the diphtheria exotoxin (DITOX), to activate pyroptosis, an inflammatory cell death mechanism alternative to apoptosis, in subcutaneous CRC tumors derived from organoids that had acquired resistance to 5-Fluorouracil by selection along successive *in vitro* passages (Serna et al., 2020). Here, we assessed whether the de-immunized T22-PE24-H6 nanotoxin could induce the activation of the pyroptotic cell death pathway and provide new possibilities in oncotherapy. We also tested if this nanotoxin was able to selectively eliminate CXCR4^+^ target cancer cells and whether this could lead to effective antitumor and antimetastatic activity.

## 2. Materials and methods

### 2.1. Protein production, purification and characterization

**Figure 1.**
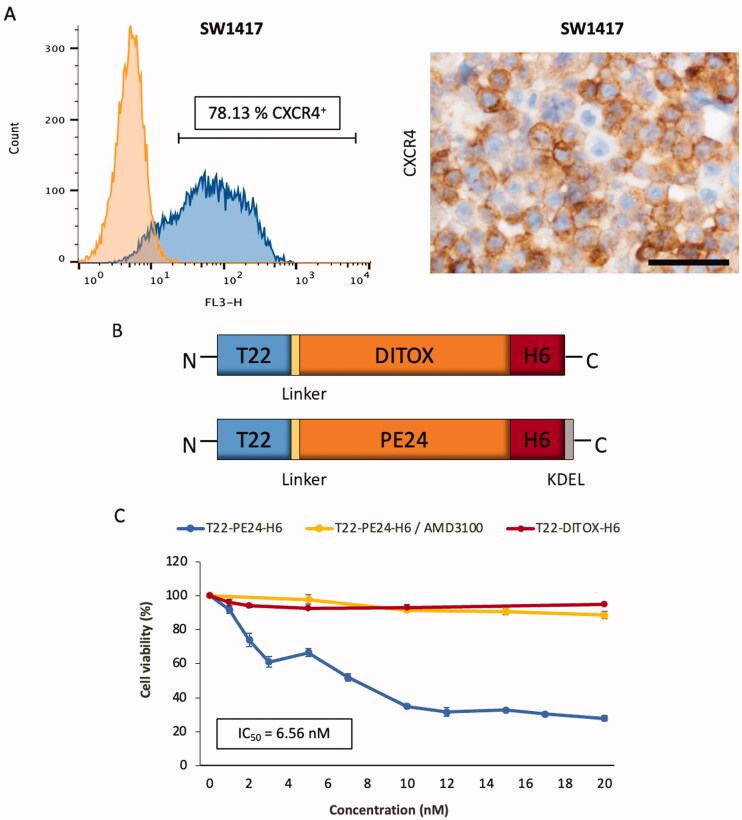
Antitumor activity of toxin-based nanoparticles in SW1417 CRC cells *in vitro*. (A) CXCR4 membrane expression of the SW1417 cell line was measured by flow cytometry or by ICC in SW1417 cell blocks. Scale bar: 50 µm. (B) Schematic representation of the polypeptidic building blocks of the T22-PE24-H6 and T22-DITOX-H6 constructs (box sizes are only indicative). Linker indicates a short peptidic spacer to ensure flexibility of the protein domains. (C) Cell viability of the CXCR4^+^ SW1417 cells upon exposure to a 0–20 nM range of T22-PE24-H6 and T22-DITOX-H6 toxin-based nanoparticles for 48 h. The selectivity of the cytotoxic effect was also studied by performing a 30 min treatment with the antagonist AMD3100 previous to nanotoxin exposure in CXCR4^+^ SW1417 cells. Data presented as mean ± s.e.m. (*N* = 2 in triplicate experiments).

Synthetic codon-optimized genes encoding for T22-DITOX-H6 and T22-PE24-H6 modular proteins were designed in-house (Figure 1B) and provided by Geneart (ThermoFisher) subcloned into a pET22b plasmid. Recombinant plasmids were then transformed into Escherichia coli Origami B (BL21, OmpT−, Lon−, TrxB−, Gor−, Novagen) and proteins were produced overnight at 20 °C upon addition of 0.1 mM of IPTG (isopropyl-β-D-thiogalactopyranoside). Bacterial cells were then harvested by centrifugation (5000 *g*, 5 min) and the obtained pellets were resuspended in wash buffer (20 mM Tris-HCl pH 8.0, 500 mM NaCl, 10 mM imidazole) in presence of complete EDTA-Free (Roche) protease inhibitors. Cell disruption was performed in an EmulsiFlex C5 cell disruptor (Avestin) at 8000–10,000 psi, soluble fraction separated by centrifugation (15,000 *g*, 45 min), and the soluble protein purified by Immobilized Metal Affinity Chromatography (IMAC) with a HisTrap HP 1 ml column (Cytiva) in an ÄKTA Pure system (Cytiva). Protein elution was achieved by a linear gradient of elution buffer (20 mM Tris, 500 mM NaCl, 500 mM Imidazole, pH = 8) and pure protein fractions were then dialyzed against a sodium carbonate solution (166 mM NaCO_3_H, pH = 8). Protein purity was determined by SDS-PAGE and Western blot using an anti-His monoclonal antibody (Santa Cruz Biotechnology) and protein integrity was verified by MALDI-TOF mass spectrometry. Finally, protein concentration was determined by the Bradford assay.

The volume size distribution of generated nanoparticles was analyzed by Dynamic Light Scattering (DLS) in a Zetasizer Pro Blue (Malvern) at 633 nm with a backscattering detector (173°). Additionally, the size and shape of nanoparticles were also evaluated at a nearly native state in a Field emission scanning electron microscope (FESEM) Zeiss Merlin (Zeiss) operating at 1KV as described in (Sánchez-García et al., 2018).

### 2.2. Cell culture and CXCR4 expression analysis

The SW1417 CRC cell line was purchased from the American Type Culture Collection (ATCC, Manassas, VA, USA) and was cultured at 37 °C and 5% CO2 in DMEM supplemented with 10% fetal bovine serum, 100 units/ml penicillin, and 100 µg/ml streptomycin (Thermo Fisher, Waltham, MA, USA). Moreover, SW1417 cells were engineered to express the luciferase reporter gene (pPK-CMV- F3, Promokine, TE Huissen, The Netherlands) by transfection with Lipofectamine 2000 (Thermo Fisher).

Before conducting all *in vitro* and *in vivo* experiments, the levels of CXCR4 expression in the cell membrane were assessed by flow cytometry. For that purpose, we labeled 1 million cells in 100 µl of PBS with 5 µl of the PE-Cy5 Mouse Anti-Human CD184 monoclonal antibody (560937, BD Biosciences, Becton Dickinson, Franklin Lakes, NY, USA) and 1 million cells in 100 µl of PBS with 5 µl of the PE-Cy5 Mouse IgG2a isotype (BD Biosciences) as a negative control. Cells were incubated in the dark for 20 minutes at 4 °C with gentle agitation, washed, and analyzed in the FACSCalibur cytometer (BD Biosciences). We measured both the percentage of the population expressing CXCR4 and the intensity of the expression expressed as mean fluorescence intensity (MFI).

### 2.3. Cytotoxic effect assays

The *in vitro* cytotoxic effect of different nanoparticles was evaluated by measuring cell viability using the XTT colorimetric cell proliferation kit II (Roche Diagnostics, Basel, Switzerland). SW1417 cells were seeded in 96 well plates and incubated O/N at 37 °C. After 24 hours, the T22-GFP-H6 nanoparticle, or its therapeutic derivatives were added at different concentration ranges [0–2 µM] for 48 hours. The competitive assays were performed by pre-treating the cells with AMD3100 (molar ratio 1 T22-PE24-H6: 10 AMD3100). Then, cells were incubated with the XTT labeling mixture for 4 hours and the formed formazan dye was quantified using a scanning multi-well spectrophotometer at 490 nm (FLUOstar OPTIMA, BMG Labtech, Ortenberg, Germany). The measured absorbance directly correlates with the number of viable cells. Data were reported as a percentage of cell viability of nanoparticle-treated cells in relation to buffer-treated cell viability.

### 2.4. Antitumor activity assessment in a subcutaneous CRC model

To assess the antitumor activity of the T22-PE24-H6 nanotoxin we used the SW1417 subcutaneous model in Swiss Nude mice. All procedures were conducted in accordance with the guidelines approved by the institutional animal Ethics Committee of Hospital Sant Pau. Swiss nude female mice (4 weeks old) were obtained from Charles River Laboratories and ten million CXCR4^+^ SW1417 cells were injected in two subcutaneous flanks. Once tumors reached approximately 120 mm^3^ sizes, mice were randomized into the control or treated group. Treated mice received intravenous doses of 10 μg of T22-PE24-H6 (*N* = 6 mice), at a repeated dose regime of three times a week, per eight doses. The control group received a buffer using the same administration schedule. Overall the experimental period, mouse body weight and tumor volume were registered three times a week. Tumor bioluminescence was also measured once a week using the IVIS Spectrum equipment (Perkin Elmer, Waltham, MA, USA). When tumors reached a volume of 600 mm^3^, mice were euthanized and tumor and non-tumor organs were collected for further analyses.

### 2.5. Antimetastatic effect assessment in an orthotopic CRC model

To assess the antimetastatic activity of the T22-PE24-H6 nanotoxin we used the metastatic SW1417 orthotopic model in 4 weeks old NSG mice (Charles River Laboratories, L'Arbresle, France). CXCR4^+^ SW1417 cells were injected into the cecum of NSG mice following the orthotopic cell microinjection (OCMI) procedure described elsewhere (Céspedes et al., 2007) and three days after, primary tumor bioluminescence was measured using the IVIS Spectrum equipment. Mice were then randomized into the control (*N* = 9) and treated (*N* = 9) group and received intravenous doses of T22-PE24-H6 at a repeated-dose regime of 5 μg, three times a week, for a total of eighteen doses. The control group received a buffer using the same administration schedule. Overall the experimental period, mouse body weight and health status were controlled three times per week. Primary tumor bioluminescence was registered once per week using the IVIS Spectrum equipment and the experiment finished when the first mouse needed to be euthanized. Necropsy and *ex vivo* imaging of the organs of interest were performed 24 hours after the last dose.

### 2.6. *In vivo* and *ex vivo* bioluminescence monitoring

*In vivo* mice, tumor progression was registered once per week by injecting intraperitoneally 2.25 mg of D-Luciferin (150 µl of the prepared 15 mg/mL stock, diluted in sterile physiological solution; PerkinElmer). After 5 minutes and under isoflurane anesthesia (XGI-8 system, Xenogen, Alameda, CA, USA), bioluminescent signals emitted by tumor cells were detected and measured using the IVIS Spectrum equipment. Registration of bioluminescence emission was performed in the mouse ventral or dorsal position depending on the cell injection site.

At the end of each experiment, a complete necropsy procedure was performed, and all relevant organs (liver, kidneys, lungs, diaphragm, spleen, pancreas, colon, intestinal tract, and mesenteric lymph nodes) were collected and covered in a 0.75 mg/kg D-Luciferin solution in 12 or 6 wells plates and imaged as well. Exposure time was adjusted automatically according to the bioluminescence intensity of each mouse and cell line. Then, the obtained images were analyzed with the Living Image Software v4.5.5 (PerkinElmer) and the photons/sec emitted by cells were quantified for each mouse or organ.

### 2.7. Histopathological analysis and metastatic foci quantitation

All relevant organs described above were paraffin-embedded and stained with H&E to perform a complete histopathological analysis and a clinical pathologist supervised all samples for toxicity evaluation. Tumor sections stained with either H&E or DAPI were also used to assess the cytotoxic effect of the administered T22-PE24-H6 nanotoxin *in vivo*, by counting the number of cell death bodies in 10 high-power fields (magnification 400x). DAPI staining was performed in Triton X-100 (0.5%) permeabilized sections mounted with DAPI mounting media (ProLong Gold antifade reagent, Thermo Fisher). Samples were evaluated under a fluorescence microscope at a wavelength of λex = 334 nm/λem = 465 nm.

In the orthotopic *in vivo* experiments, the spreading of cells from the primary tumor (colon) to other distant organs was analyzed in H&E-stained samples. We studied those organs in which metastatic dissemination is expected in CRC: liver, lung, mesenteric lymph nodes, and peritoneum. An Olympus microscope with the Cell^D Olympus software was used to count the number and measure the size (expressed as µm^2^) of all observed metastatic foci in three different slices of each organ.

### 2.8. Immunocytochemistry and immunohistochemistry

The study of cell death pathways induced by the T22-PE24-H6 was performed both in *in vitro* treated cells aggregated in cell blocks and in the subcutaneous CRC tumors of treated mice. SW1417 cells were treated with 6 nM of T22-PE24-H6 for 2, 5, 24, and 48 hours and then trypsinized and centrifuged. The obtained pellet was mixed with plasma and thrombin (Sigma Aldrich, San Luis, MO, USA) and quickly agitated to form a clot. The clot was placed in a tissue cassette, fixed with formaldehyde, and then paraffin-embedded for further immunocytochemistry (ICC) analysis. Markers of apoptosis such as active caspase-3 protein (early apoptosis, 1:300, C92-605, BD Bioscience) or proteolyzed PARP (late apoptosis, 1:300, g7341, Promega, Madison, WI, USA) or pyroptosis such as NLRP3 (1:300, AG-20B-0014-C100, Adipogen, San Diego, CA, USA), cleaved caspase-1 (1:400, PA5-99390, Thermo Fisher) and cleaved GSDMD (1:500, 36425, Cell Signaling, Danvers, MA, USA), were assessed. The percentage of stained surface and its intensity were calculated using the ImageJ software and the Color Deconvolution Plugin with the H DAB vector to split the brown staining adjusting the threshold to each marker. Then, the Analyze particles plugin was used to detect all stained areas and the mean gray value was obtained by combining all selected black areas. The intensity value was calculated by subtracting to 255 the mean gray value obtained in the analysis. Up to 5 high-power fields (400x) of each sample were analyzed and the results were expressed as mean ± s.e.m.

We have also used IHC in the tissue samples obtained from *in vivo* experiments to assess the expression of CXCR4 (1:200, ab124824, Abcam, Cambridge, UK) in subcutaneous tumors, CRC primary tumors, and metastases located in distal organs.

### 2.9. Statistical analysis

*In vitro* cell, viability assays were performed in biological triplicates and the data were expressed as mean ± standard error of the mean (s.e.m.). IC_50_ was calculated with SigmaPlot (Systat Software, Inc) using a non-linear regression test with Hill-3 parameter adjustment. In the *in vivo* experiments, we analyzed differences in the number of metastatic foci and foci size between buffer-treated vs nanoparticle-treated mice, using the Mann-Whitney U test, since data was obtained for all measured variables did not follow a normal distribution. Results on the number of mitotic figures, number of cell death bodies, and active caspase 3, proteolyzed PARP, cleaved caspase-1, and cleaved GSDMD stained cells between groups were analyzed using both the Student’s *t*-test and the Mann-Whitney U test. Data were reported as mean ± s.e.m. and differences between groups were considered significant at *p* < .05. The differences between relevant data were indicated as * for statistical significance among the designated groups. Statistical calculations were performed using GraphPad Prism 8 software.

## 3. Results

### 3.1. Highly potent and CXCR4-dependent T22-PE24-H6 cytotoxic effect in CXCR4^+^ CRC cells

We first confirmed the high expression of the CXCR4 receptor in SW1417 CRC cells by flow cytometry and immunocytochemistry (Figure 1A). Following, we tested the activity of two different previously generated toxin-based nanoparticles (Sánchez-García et al., 2018), which structure is graphically described in Figure 1(B), in CXCR4^+^ SW1417 cells *in vitro*. In short, T22-PE24-H6 is a modular protein that contains the de-immunized catalytic domain of the *Pseudomonas aeruginosa* exotoxin A fused to the CXCR4-specific ligand T22 at the amino-terminal and a poly-histidine tag at the carboxy-terminal. It also contains a KDEL sequence to induce its cytosolic release via retrograde transport through the secretory pathway. On the other hand, T22-DITOX-H6 is an equivalent construction that contains the catalytic domain of the diphtheria toxin from *Corynebacterium diphtheriae* together with its natural translocation domain for efficient cytosolic release (Sánchez-García et al., 2018). Both modular proteins contain a furin-cleavable site between T22 and the catalytic domain to release the amino-terminal peptide once internalized. Finally, as described before, both proteins self-assemble into pseudo-spherical nanoscale structures with average sizes around 40 nm as determined by DLS and FESEM (Sánchez-García et al., 2018). We surprisingly found that CXCR4^+^ SW1417 cells were not sensitive to T22-DITOX-H6 nanoparticles (Figure 1C). In contrast, CXCR4^+^ SW1417 cell exposure to a concentration range of 0–20 nM of T22-PE24-H6 nanotoxin, showed a very potent and dose-dependent antineoplastic effect, with an IC_50_ of 6.56 nM (Figure 1C). Moreover, competition assays using CXCR4^+^ SW1417 cells pretreated with the CXCR4 inhibitor AMD3100, showed a dramatic reduction in T22-PE24-H6 anticancer activity, preventing the nanotoxin-induced cell death and maintaining cell viability close to 100% (Figure 1C). Thus, the T22-PE24-H6 nanotoxin displays a potent and completely CXCR4-dependent cytotoxic effect in CXCR4^+^ SW1417 cells *in vitro*.

### 3.2. T22-PE24-H6 repeated dose administration reduces tumor volume without toxicity in non-target organs

We next evaluated the antitumor effect of the T22-PE24-H6 and whether the induction of cell death observed *in vitro*, could be replicated in *in vivo* models. Thus, CXCR4^+^ SW1417 cells were injected into Swiss nude mice to generate a subcutaneous tumor model. When tumor size reached an approximately 120 mm^3^ of size, mice were randomized into two groups (*N* = 6) that were administered with a repeated dosage of 10 µg T22-PE24-H6 or buffer, three-time a week, per 8 doses (which we had previously observed not to produce any toxic effect in non-target organs in the same mouse strain).

**Figure 2.**
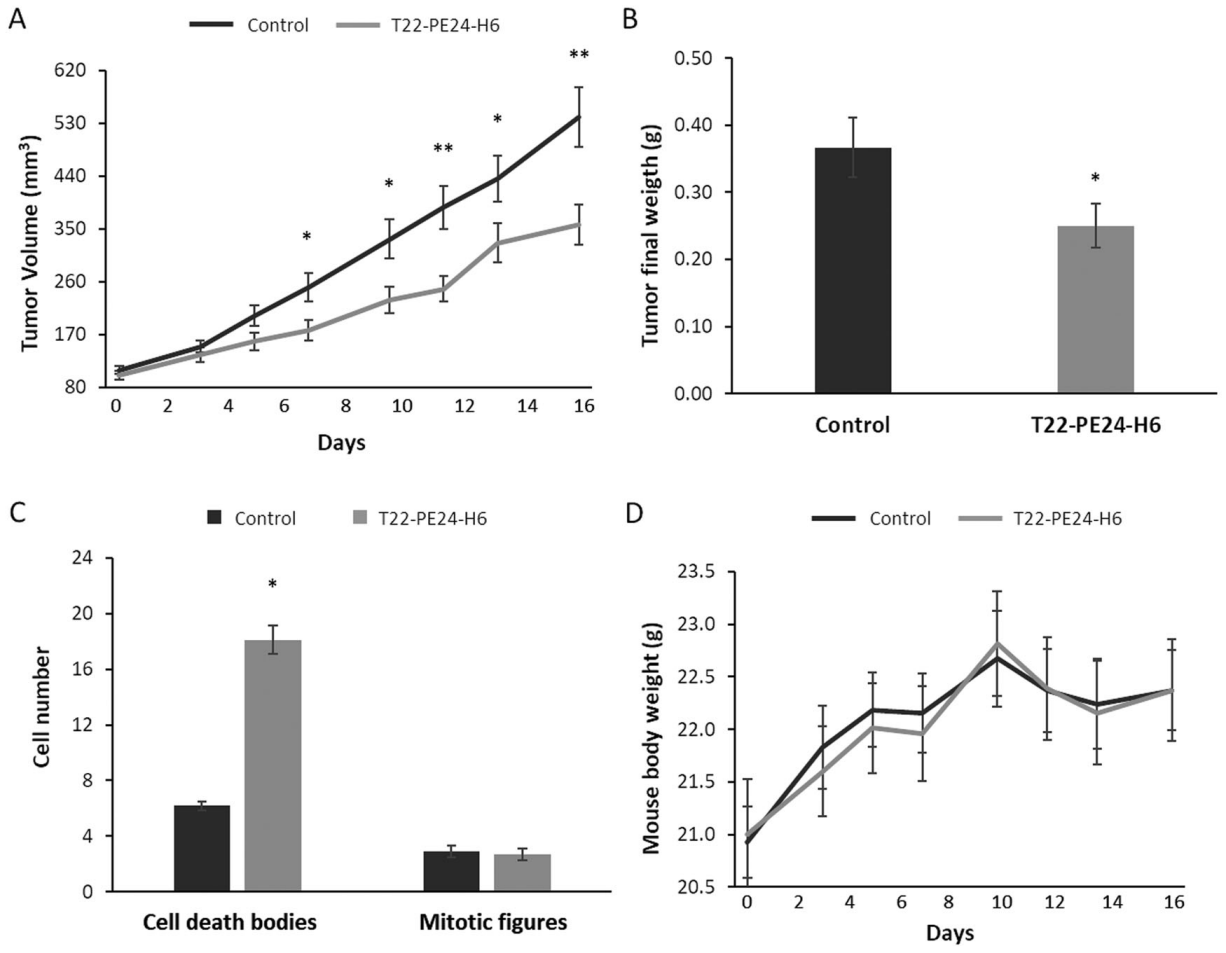
T22-PE24-H6 antitumor effect in a cell-derived subcutaneous SW1417 CRC model. (A) Antitumor effect of T22-PE24-H6 measured by the reduction of tumor volume (mm^3^). (B) Antitumor effect of T22-PE24-H6 measured by tumor weight (g) at the end of the experiment. (C) Increase in the number of cell death bodies in the SC SW1417 tumors counted in 10 high-power fields of H&E-stained tissue samples collected at the end of the experiment. (D) Follow-up of mouse body weight (g) during the repeated dose administration of 10 µg T22-PE24-H6 (three times a week, 8 total doses). All data are presented as mean ± s.e.m., *N* = 6. *p < 0.05; **p < 0.01.

After only four T22-PE24-H6 doses, significant differences (*p* = 0.02) in tumor volume were already detected between control (buffer-treated) and T22-PE24-H6-treated mice (Figure 2A). Moreover, at the end of the experiment, after the eighth dose, we observed a 1.6-fold reduction in tumor volume, as compared to buffer-treated mice (*p* = 0.005), which was associated with a significant reduction in final tumor weight (*p* = 0.04) (Figure 2B). This antitumor effect was a likely consequence of the 3.0-fold increase in the number of cell death bodies observed in tumor tissue (*p* < 0.05) (Figure 2C). Moreover, at the end of the experiment, nanotoxin-treated and buffer-treated mice did not show significant differences in mouse body weight (Figure 2D). The histology of non-target organs, such as the liver and kidneys, was evaluated by H&E-stained sections, observing a complete lack of histological alterations in these tissues.

### 3.3. Lack of apoptosis activation after T22-PE24-H6 nanotoxin treatment

**Figure 3.**
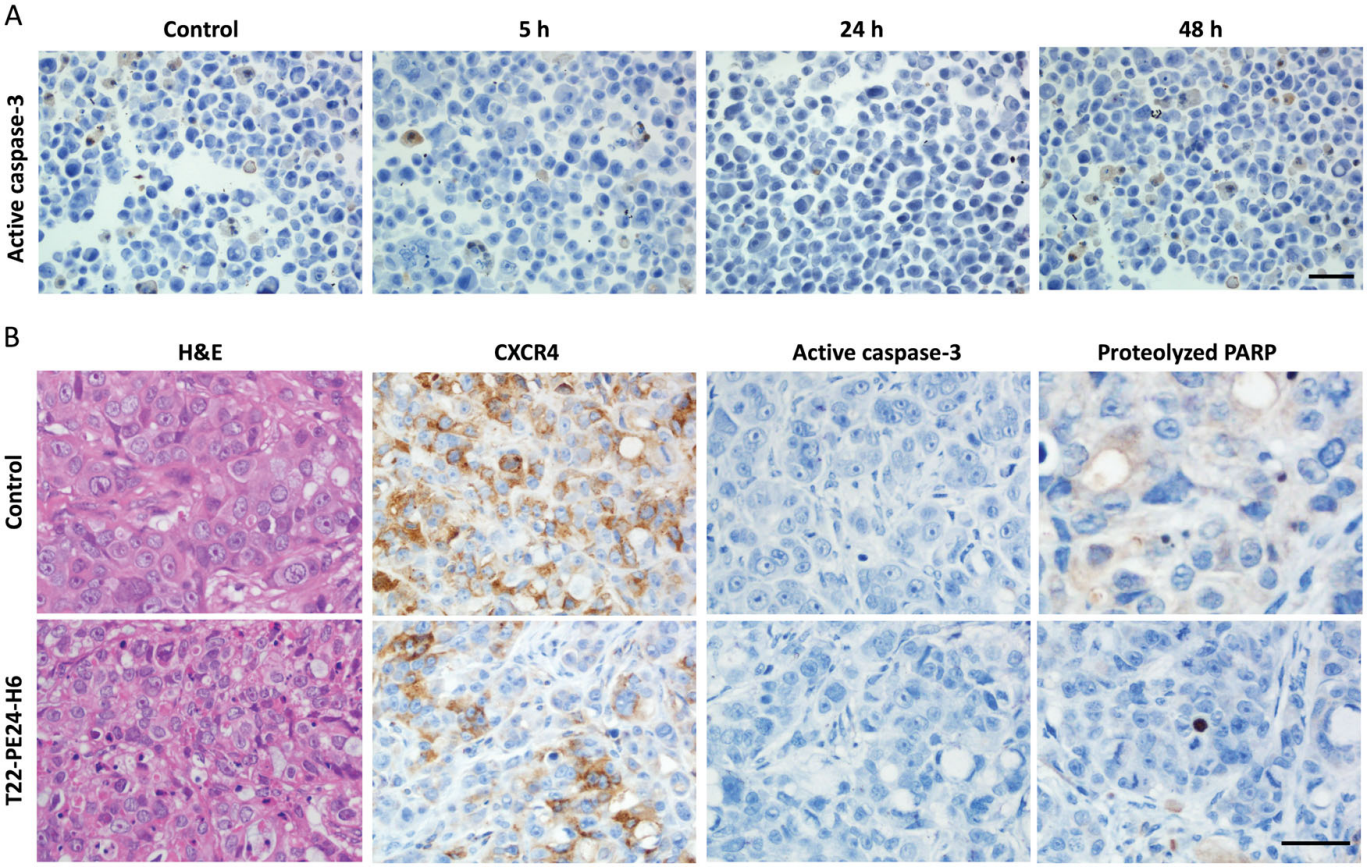
*In vitro* and *in vivo* assessment of apoptosis activation after T22-PE24-H6 treatment. (A) Representative images of ICC staining of active caspase-3 in cell blocks of CXCR4^+^ SW1417 cells exposed to 6 nM T22-PE24-H6 for 5, 24 or 48 h. Stained sections were compared to untreated control CXCR4^+^ SW1417 cells. (B) Representative images of H&E and IHC staining of CXCR4, active caspase-3 and proteolyzed PARP in SW1417 subcutaneous tumors from Swiss nude mice treated with buffer or 10 µg of T22-PE24-H6, three times a week and 8 total doses. Scale bars: 50 µm.

We previously reported that the diverse therapeutic nanoparticles, either protein-only nanoparticles or nanoconjugates developed so far in our group, containing different cytotoxic targets (DNA, microtubules, or mitochondrial proteins), kill cancer cells mainly by apoptosis or mitotic catastrophe. Thus, we next determined whether the potent antitumor effect of the T22-PE24-H6 nanotoxin was mediated by activation of the classical apoptotic cell death pathway. For that purpose, CXCR4^+^ SW1417 cells were cultured and exposed to the IC_50_ of T22-PE24-H6 (6 nM) at different exposure times (2, 5, 24, or 48 hours). Control CXCR4^+^ SW1417 cells were also cultured without being exposed to the nanotoxin. The immunocytochemical assessment of the expression of active caspase-3 yielded a lack of differences in staining between control and nanotoxin-treated cells (Figure 3A).

We also studied apoptotic activation in the CXCR4^+^ SW1417 subcutaneous tumors after treatment with eight 10 µg T22-PE24-H6 doses, in which we had found a 3-fold increase in the number of cell death bodies and a decrease in the expression of CXCR4. By immunohistochemistry against apoptotic markers in tumor tissue sections, we found no detectable or very low signal of active caspase-3 or proteolyzed-PARP staining in nanotoxin-treated tumors (Figure 3B). These results suggested that the mechanism of cell death was not mediated by apoptosis induction.

### 3.4. T22-PE24-H6 induction of the pyroptotic cell death pathway

**Figure 4.**
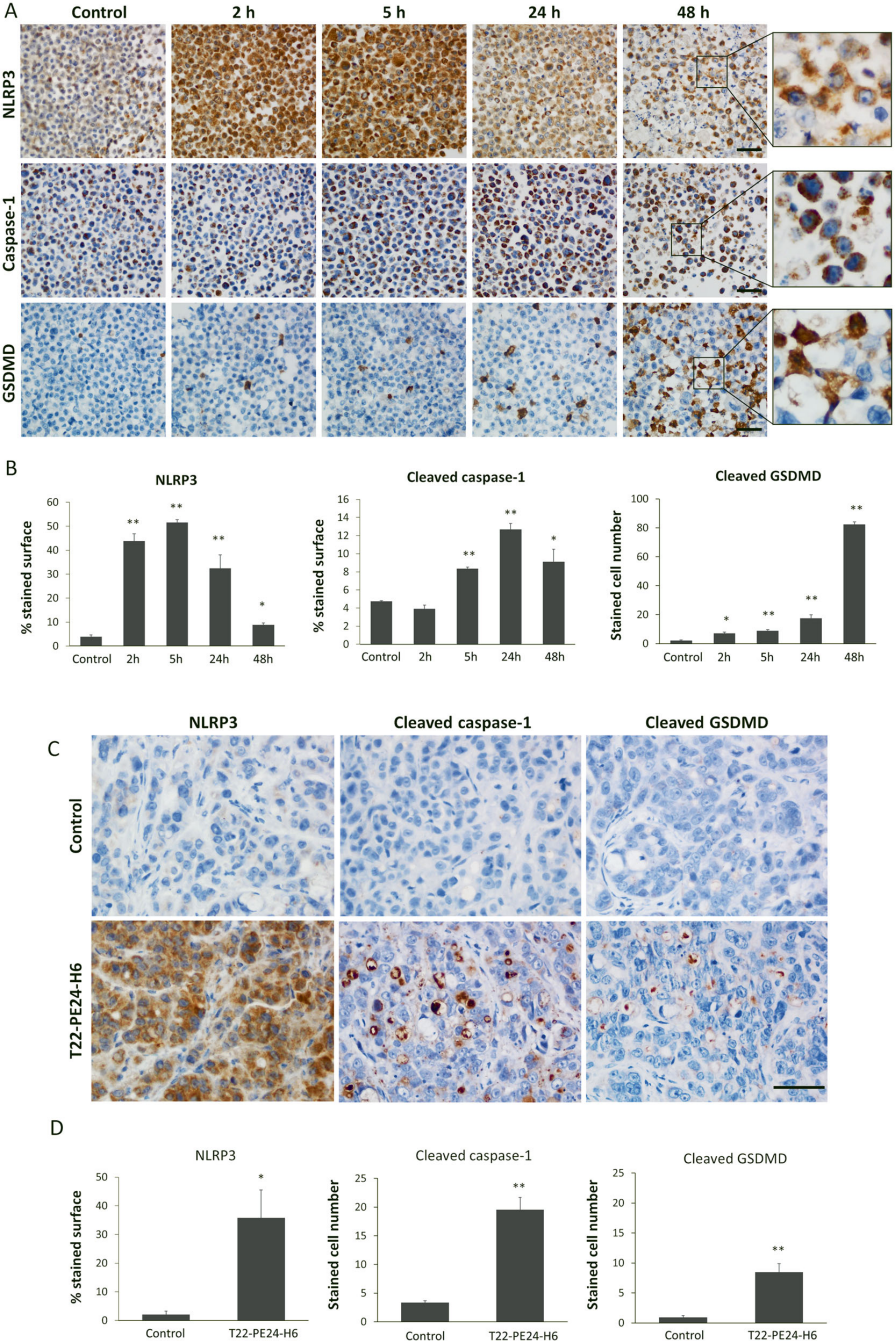
Activation of the pyroptotic pathway in CXCR4^+^ SW1417 cultured cells and in subcutaneous tumors after T22-PE24-H6 treatment. (A) Detection of pyroptotic markers, including NLRP3, cleaved caspase-1 and cleaved GSDMD, by ICC in cell blocks of CXCR4^+^ SW1417 cells exposed for 2, 5, 24 or 48 h to 6 nM of T22-PE24-H6. Stained sections were compared to untreated control CXCR4^+^ SW1417 cells. Scale bars: 50 µm. (B) Quantitation values of each marker staining in SW1417 control cells or in cells treated with T22-PE24-H6. In ICC of NLRP3 and caspase-1, up to 5 high-power fields (400x) were imaged and the percentage of stained surface was measured with ImageJ. In GSDMD staining, up to 5 high-power fields (400x) were analyzed by counting the number of GSDMD positive cells. (C) Representative images of IHC staining of NLRP3, cleaved caspase-1 and cleaved GSDMD in SW1417 SC tumors from Swiss nude mice treated with 10 µg of T22-PE24-H6, three times a week and 8 total doses. Scale bars: 50 µm. (D) Quantitation of pyroptotic markers in tumors from buffer-treated and T22-PE24-H6-treated mice. NLRP3 staining (5 high-power fields (400x) analyzed with ImageJ) was expressed as a percentage of the stained surface. To assess caspase-1 and GSDMD stained sections, we instead counted the number of positive cells in 5 high-power fields (400x). Measurements in tissue sections were performed 24 h after the last administered dose, being compared to buffer-treated control mice. All the data are expressed as mean ± s.e.m. * *p* < 0.05; ** *p* < 0.001.

Since we could not detect activation of the apoptotic pathway after T22-PE24-H6 therapy, both *in vitro* and *in vivo*, we explored possible alternative mechanisms of cell death. Pyroptosis has been recently described to be triggered by epithelial cells upon infection by intracellular bacterial pathogens (Jorgensen & Miao, 2015). Although this mechanism of action has been barely exploited in cancer therapy, we have recently reported the T22-DITOX-H6 induction of pyroptosis in tumors unable to induce apoptosis (Serna et al., 2020). Therefore, we analyzed whether the protein-only nanoparticle T22-PE24-H6, incorporating the PE24 exotoxin, was capable to induce pyroptosis in our CRC models, by determining the activation of different molecular components of this pathway after exposure to this nanotoxin *in vitro* or after its intravenous injection in mice *in vivo* (Figure 4).

After exposure of CXCR4^+^ SW1417 cells to the T22-PE24-H6 nanotoxin for a short time, at 2 or 5 hours, we found in almost all cancer cells a high overexpression of the NLRP3 protein, an inflammatory marker that initiates the signaling cascade that leads to pyroptosis. This expression was maintained in a reduced percent of cancer cells during the 24–48 hours period, which however continued being expressed at a high intensity (Figure 4A and B). We next observed that the activation of the NLRP3 inflammasome was subsequently followed by the generation of active caspase-1 at longer exposure times (5, 24, and 48 hours), which in turn, cleaved the pro-Gasdermin D (pro-GSDMD) protein. It is known that the active GSDMD N-terminal fragment released after cleavage translocates to the plasma membrane, where it oligomerizes to form pores in the cell surface (Weldon & Pastan, 2011; Mulvihill et al., 2018). Consistently, we observed a highly evident GSDMD translocation to the membrane of CXCR4^+^ SW1417 cells with very high intensity in 10–20% of exposed cancer cells, mainly after 48 hours of exposure to T22-PE24-H6 (Figure 4A).

The sequential activation of highly expressed pyroptotic markers was also observed in T22-PE24-H6-treated CXCR4^+^ SW1417 tumors, as compared to their negligible expression in buffer-treated tumors. Thus, NLRP3, cleaved caspase-1, and cleaved Gasdermin D (GSDMD) showed a higher expression 24 hours after the last dose (Figure 4C), in the repeated T22-PE24-H6 nanotoxin dosage administration described above, which previously showed significant antitumor activity (Figure 2). Again, the first marker to be activated in tumor tissues was NLRP3 with a cytoplasmic expression pattern in most of the tumor cells. After that, caspase-1 is activated showing very specific staining in the subset of cancer cells undergoing cell death, which increased 6-fold the number of stained cells as compared to buffer-treated tumors. Finally, GSDMD was also activated in the pyroptotic bodies causing pore formation and cell death, according to its role as a pyroptotic cell effector (Figure 4D).

### 3.5. Selective pyroptotic effect on CXCR4^+^ cancer cells based on T22-PE24-H6 multivalency

**Figure 5.**
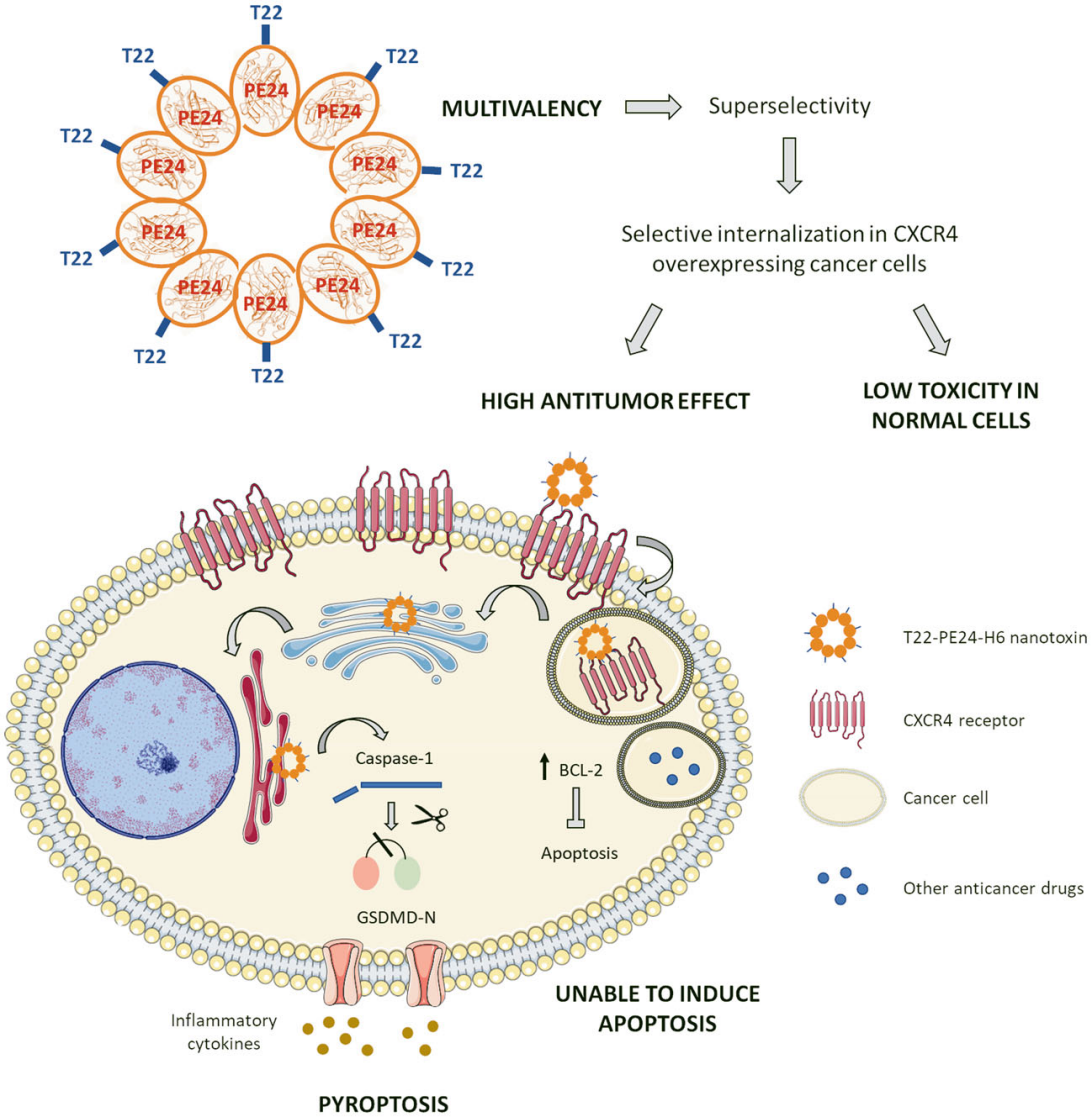
Graphical summary showing the T22-PE24-H6 highly selective antitumor effect in CXCR4 overexpressing CRC cells, through the activation of pyroptosis. The image describes the advantage of this novel approach in front of other anticancer therapies, due to the super selectivity of the polytpeptidic nano toxins that allows its specific internalization in CXCR4^+^ target cancer cells and the induction of pyroptosis, a non-apoptotic cell death mechanism. T22-PE24-H6 interacts with the CXCR4 receptor in the cell surface of cancer cells, being subsequently internalized by endocytosis and trafficked through the Golgi apparatus and the endoplasmic reticulum (ER). Once in the cytoplasm, the *Pseudomonas aeruginosa* exotoxin is able to induce target cancer cell death by activation of the pyroptotic effectors NLRP3 (part of the inflammasome complex), caspase-1 and Gasdermin-D (GSDMD). The activation of this novel mechanism will overcome resistance to classical anticancer drugs mediated by cancer cell upregulation of antiapoptotic pathways (e.g. overexpression of Bcl-2 protein family).

The integration of the previously described structural information on the T22-PE24-H6 self-assembling nanoparticle and the antitumor effect observed in this work, which is mediated by the activation of the pyroptotic cell death pathway, leads us to propose a mechanistic view of this novel therapeutic approach (Figure 5) that could overcome, at least in part, the relapse and metastatic dissemination that occurs when resistance to classical chemotherapy develops, in CRC and in other tumor types. Thus, the fact that the homo-oligomeric T22-PE24-H6 nanoparticle displays on its surface several copies of the CXCR4 ligand T22 (estimated to be 10–12 in a very similar construct) (Rueda et al., 2015), and that CXCR4^+^ tumors in CRC patients show an overexpression of the CXCR4 receptor (as compared to normal tissues, including the CXCR4^+^ bone marrow cells) (Serna et al., 2020), as it happens with the CXCR4^+^ SW1417 model that we here used (Figure 1A), makes the delivery of exotoxin cytotoxic domain in the target cell cytosol of high selectivity compared with the negligible delivery of this domain in normal cells (Falgàs et al., 2020). This would lead to a high antitumor effect in tumor tissue and a lack of toxicity in non-tumor organs (Figure 5).

On the other hand, the lack of induction of apoptosis by the T22-PE24-H6 in CXCR4^+^ CRC cells, both *in vitro* and *in vivo*, and the capacity of this nanotoxin to sequentially activate the pyroptotic markers NLRP3, caspase-1, and GSDMD, in CXCR4^+^ CRC cells, leading to its selective and CXCR4-dependent cell death, would establish this novel nanotechnological anticancer approach as a powerful alternative for the treatment of CRC tumors once they develop relapse and metastasize, especially, when recurrence is due to the upregulation of anti-apoptotic proteins (Hanahan & Weinberg, 2011) (Figure 5).

### 3.6. T22-PE24-H6 prevents the development of lymphatic and hematogenous metastases in the CXCR4^+^ SW1417 cell-derived CRC model

Once we demonstrated that the T22-PE24-H6 nanotoxin has a potent antitumor effect, mediated by pyroptotic cell death induction, in CXCR4^+^ SW147 subcutaneous tumors, we performed an experiment to determine its capacity to prevent cancer cell dissemination and metastatic foci growth at the clinically relevant organs affected by metastases in an orthotopic CXCR4^+^ SW1417 CRC model. This model metastasizes to lymph nodes, liver, lung, and peritoneum. We expected that treatment with a T22-PE24-H6 repeated dosage could reduce the number and size of metastatic foci at the end of the experiment. For that purpose, NSG mice were implanted in the cecum with two million CXCR4^+^ SW1417 CRC cells. After three days, we started administering the nanotoxin to the mice in the experimental group, following the same repeated-dose schedule used for antitumor treatment, or with buffer in the control group. Treatment continued until the first mouse, belonging to either the control or treated group, achieved the criteria for euthanasia.

**Figure 6.**
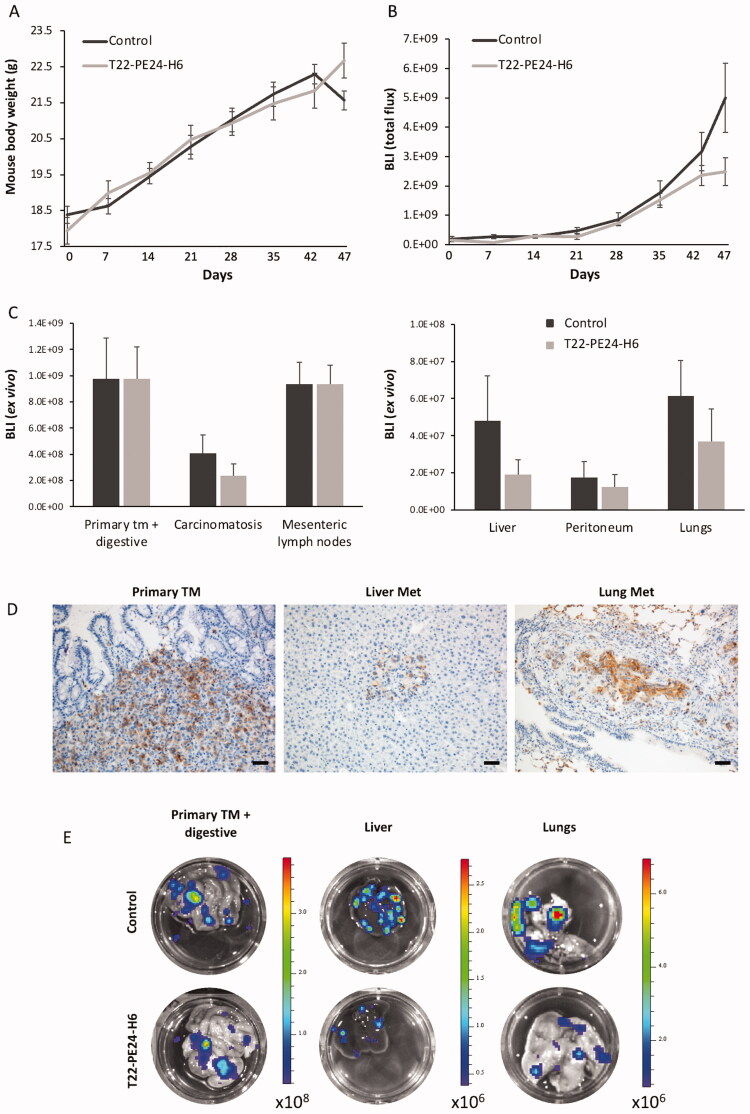
T22-PE24-H6 antimetastatic effect in the cell-derived orthotopic CXCR4^+^ SW1417 CRC model. (A) Evolution of mouse body weight (g) during the repeated dose administration of 5 µg of T22-PE24-H6 (three times a week, 18 total doses). (B) Total body bioluminescence (BLI; Total Flux [p/s]) of buffer and T22-PE24-H6 treated mice, was measured once per week throughout the experiment. (C) Comparison of bioluminescence emission between the buffer-treated and the 5 µg T22-PE24-H6 repeated-dose schedule groups in the primary tumor, mesenteric lymph nodes, liver, lung, and peritoneum. Results are presented as mean ± s.e.m. bioluminescence values in photons per second (total flux [p/s]). (D) CXCR4 IHQ images show that the receptor is highly expressed in cancer cells from the primary tumor, liver, and lung metastasis. Scale bar: 50 µm. (E) Representative bioluminescence images comparing primary tumor and metastatic dissemination in liver and lungs of mice treated with the nanotoxin (5 µg T22-PE24-H6) or buffer (control).

After 18 doses, control mice started to lose weight due to metastatic dissemination, a time point at which all mice were euthanized (Figure 6A). At this point, we could find differences close to significance in total body bioluminescence, emitted by luciferase-expressing cancer cells, between mice treated with T22-PE24-H6 and buffer-treated mice (Figure 6B). The *ex vivo* bioluminescence analysis of each cancer-affected organ showed differences in signal between both groups (Figure 6C and E). The bioluminescence emitted by the tested organs of the nanotoxin-treated mice, which included metastatic foci disseminated to the peritoneum, liver, and lung, which maintained the expression of the CXCR4 receptor (Figure 6D), was in all cases lower than that emitted by the same organs of buffer-treated mice, which correlates with a reduced metastatic load.

Following, a histological evaluation of the metastatic load after T22-PE24-H6-treatment was performed by quantifying the developed metastatic foci and size for each affected organ in H&E-stained sections. Mice treated with T22-PE24-H6 presented a reduction in metastatic load (total metastatic area) in all affected organs, especially evident in lymph nodes (*p* < 0.05) (data not shown). Compared to the findings in the buffer-treated group, T22-PE24-H6 treatment potently prevented lymphatic (Lymph node Mets) and hematogenous (Liver Mets and Lung Mets) metastasis development, whereas its capacity to prevent transcelomic metastases (Peritoneal Mets) were lower. Consistent with the *ex vivo* bioluminescence results, mice treated with T22-PE24-H6 showed a 2-fold reduction in mean metastatic foci number in lymph node (*p* = 0.008), a 2.1-fold reduction in the liver (*p* = 0.007) and a 2.5-fold reduction in the lung (*p* = 0.01), as compared to metastatic foci number of the corresponding sites in buffer-treated mice (Table 1). There was also a trend of a reduction of the mean number of peritoneal metastatic foci in T22-PE24-H6-treated mice (similar to the reduction in bioluminescence emission by peritoneal metastases described above); however, it did not reach statistical significance. Thus, the nanotoxin reduces mainly the number of metastatic foci rather than their size, since in T22-PE24-H6 treated mice, primary tumor, lymph node, and lung metastases despite of showing a trend toward a smaller mean size, they did not achieve statistically significant differences.

**Table 1.** T22-PE24-H6 antimetastatic effect measured by prevention of metastases development in the CXCR4^+^ SW1417 cell-derived CRC metastatic model.

T22-PE24-H6 prevention of metastasis					
SW1417 cell-derived orthotopic model					
Group	Primary tumor	Lymph node Mets	Liver Mets	Lung Mets	Peritoneal Mets
	Mice %	Mice % # foci	Mice % # foci	Mice % # foci	Mice % # foci
Buffer	9/9 100%	100%	100%	100%	89%
		7.1 ± 1.1^a^	15 ± 1.9^b^	49.2 ± 9.6^c^	4.4 ± 0.7
T22-PE24-H6	9/9 100%	100%	100%	89%	89%
		3.4 ± 0.5^a^	7.1 ± 1.6^b^	19.7 ± 4.1^c^	3.2 ± 0.9
Metastatic foci size (µm² × 10^3^)					
Group	Primary tumor	Lymph nods Mets	Liver Mets	Lung Mets	Peritoneal Mets
Buffer	9428.8 ± 1081.1	739.8 ± 94.6	15.7 ± 3.5	44.1 ± 9.1	2423.4 ± 1141.2
T22-PE24-H6	7331.5 ± 978.8	513.8 ± 99.3	16.5 ± 4.2	36.1 ± 8.9	2579.2 ± 1345.9

Mean + s.e.m. metastatic foci number or area (µm^2^) per mouse, counted in three entire histology sections.

^a^*p* = 0.008; ^b^*p* = 0.007; ^c^*p* = .001.

### 3.7. Lack of toxicity of T22-PE24-H6 repeated administration in normal and metastasis-affected organs

**Figure 7.**
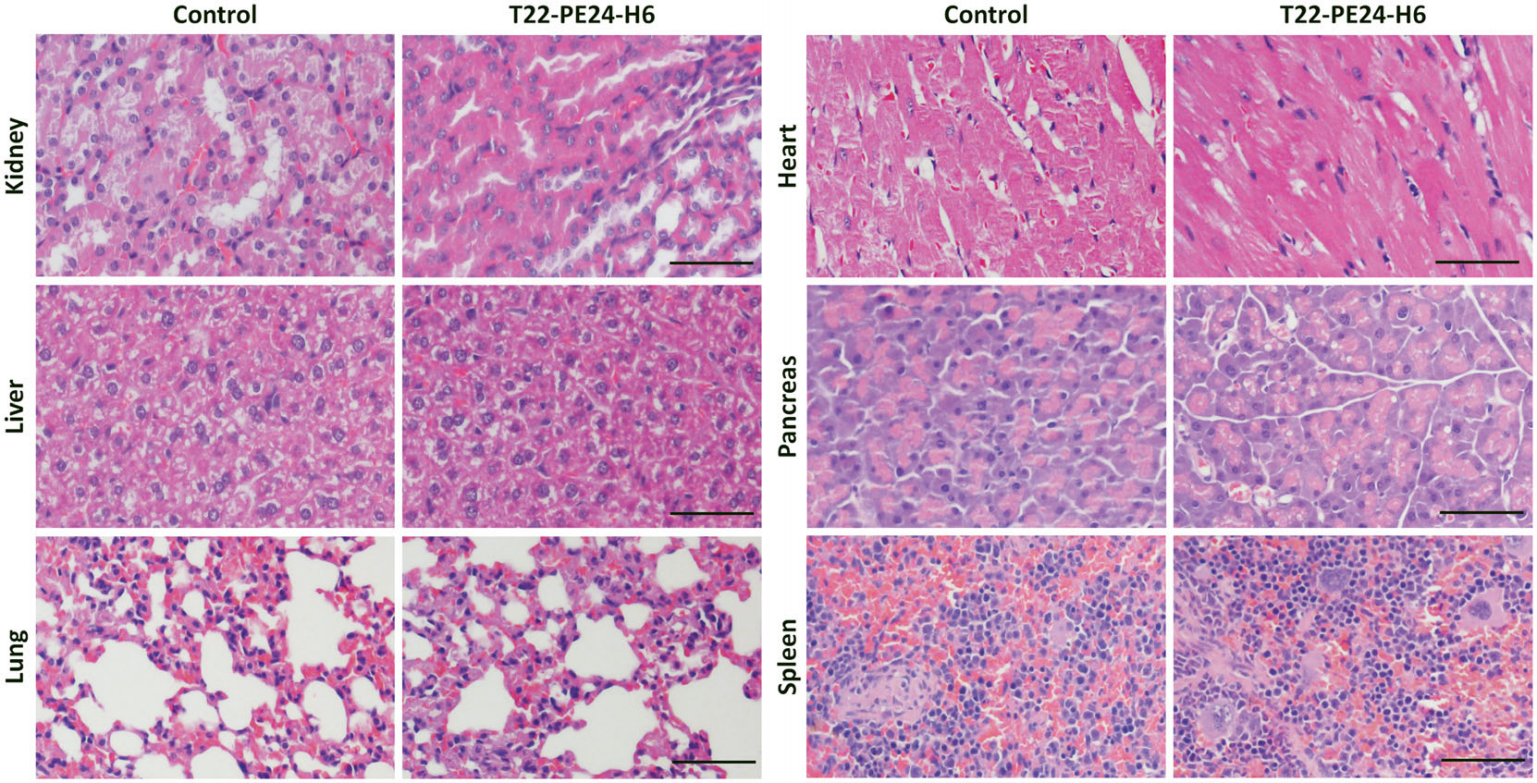
Lack of histological alterations in normal and colonized organs of mice treated with T22-PE24-H6. Representative images of H&E staining in normal organs (kidney, heart, pancreas, and spleen) and in metastasis-affected organs (liver and lung). These organs were collected 24 hours after the last buffer or T22-PE24-H6 administered dose of the repeated administration schedule (5 µg of T22-PE24-H6, 18 total doses, three times per week) in NSG mice orthotopically injected with the CXCR4^+^ SW1417 CRC cell line. Scale bars: 100 µm.

We also evaluated the effect of the T22-PE24-H6 nanotoxin in normal and metastasis affected organs in which we had previously described a transient accumulation in biodistribution assays with the T22-GFP-H6 nanoparticle by measuring fluorescence emission, because of the presence of fenestrated vessels (Céspedes et al., 2016). We principally examined for possible histological alterations in the kidneys and liver, the two organs with higher nanoparticle biodistribution, but we also analyzed all described relevant normal organs. No differences in histology were observed between control buffer-treated mice and T22-PE24-H6-treated mice in these organs. This lack of toxicity was previously found in the same organs, including normal colon, kidney, liver, and spleen or bone marrow, in a lymphoma model (Falgàs et al., 2020) and in a head and neck cancer model (Rioja-Blanco et al., 2022) treated with the same T22-PE26-H6 dosage. Consequently, the glomeruli and surrounding renal tubules were clearly visible and presented no cytoplasmic vacuolation or eosinophilic protein accumulation. In liver tissue, the hepatocytes did not lose their architecture and did not present steatosis or any other histological alteration (Figure 7). Therefore, consistently with the negligible nanoparticle biodistribution to normal tissues, the lack of histological alterations in all analyzed tissues, including also the lung, heart, pancreas and spleen, and the lack of mouse body weight loss during the repeated administration of T22-PE24-H6 (Figure 2D and 6A), indicate a wide therapeutic index for the T22‐PE24-H6 nanotoxin at a dosage that achieves a potent antimetastatic effect.

## 4. Discussion

Among all cytotoxic domains found in organisms, bacterial toxins are already being used in new strategies for cancer therapy, exhibiting a highly potent anticancer effect (Weldon & Pastan, 2011). On this basis, we previously developed protein-only nanoparticles, incorporating the de-immunized version of the catalytic domain of the exotoxin A from *Pseudomonas aeruginosa* together with a polypeptide containing the T22 peptide and a His-tag; thus, generating the self-assembling multivalent T22-PE24-H6 nanotoxin (Sánchez-García et al., 2018). This novel protein-only nanoparticle approach allowed us to achieve a faster production process by skipping the conjugation step needed for the therapeutic nanoconjugates synthesis (Sharma et al., 2012) while reducing its immunogenicity due to the replacement of the exogenous GFP protein, found in our previously developed nanoconjugates (Céspedes et al., 2018; Pallarès et al., 2020).

Our results demonstrate that the T22-PE24-H6 nanotoxin displays a potent CXCR4-dependent cytotoxic effect in CRC cells and also that its use at low doses in a repeated treatment regime is capable of inhibiting tumor growth and metastasis development without associated systemic toxicity. Thus, exposure of the CXCR4^+^ SW1417 cell line to the toxin, showed a reduction in cell viability and a lack of antitumor activity after competition with the AMD3100 antagonist, showing a highly selective CXCR4 receptor-dependent cell death induction. Furthermore, T22-PE24-H6-treated mice bearing subcutaneous tumors underwent a 1.6-fold reduction in tumor volume and a 3-fold increase in tumor cell death body induction at the end of the experiment, compared to buffer-treated mice. Moreover, when repeatedly administering low doses of T22-PE24-H6 in the highly metastatic cell-derived orthotopic model, we observed a significant 2-fold reduction in the number of lymphatic and hematogenous metastatic foci and a decreasing trend in the primary tumor and metastatic foci size. Consistently with these results, our group also proved the T22-PE24-H6 capacity to block dissemination in a diffuse large B-cell lymphoma (DLBCL) model without associated toxicity, by the elimination of CXCR4^+^ cancer cells through apoptosis induction (Falgàs et al., 2020), a result consistent with the pharmacokinetics of T22-P26-H6 that show a fast biodistribution in the bloodstream followed by a slow elimination phase having a half-life of 30 hours (Céspedes et al., 2007).

The success of this new approach relies again on achieving highly selective targeted drug delivery by exploiting the CXCR4 membrane overexpression of CRC cells as compared to normal tissues but also due to T22-PE24-H6 multivalency since it leads to super selectivity in target cell internalization (Liu et al., 2020). Also importantly, because of the capacity of the PE24 toxin to trigger the mechanism of pyroptosis, a cell death alternative to apoptosis. Thus, we found that target cell death induction by the T22-PE24-H6 nanotoxin in *in vitro* and *in vivo* CRC models is activated through the following ordered steps (Figure 5): 1) the nanotoxin, through its multiple T22 ligands, binds to CXCR4 receptors exposed in the membrane of target cells and internalizes by endocytosis, 2) furin-cleavage sites inserted between the T22 peptide and the cytotoxic PE24 domain allow its cleavage and the intracellular release of the toxin active fragment, 3) the carboxyl-terminal KDEL sequence in PE24 domain increases the efficiency of retrograde transport through the endoplasmic reticulum and its subsequent release into the cytosol and 4) the released catalytic domain will block protein synthesis by inhibition of the elongation factor 2 (EF2) to induce the activation of pyroptosis, a scarcely explored non-apoptotic cell death mechanism in cancer therapy (Tait et al., 2014; Nagarajan et al., 2019).

Importantly, the finding of T22-PE24-H6 pyroptotic induction, mediated by activation of the inflammosome marker NLRP3, followed by caspase-1 cleavage and GSDMD cleavage that translocate to the membrane in CRC models, in the absence of apoptosis activation (in sharp contrast with the observation of potent apoptosis induced by the same nanotoxin in a DLBCL lymphoma model (Falgàs et al., 2020) reinforces its use to induce anticancer activity in tumors with inactivated apoptotic pathways, at least in CRC and other solid tumors, especially after recurrence or active metastatic dissemination. It is also highly relevant to point out that T22-PE24-H6 induces a mechanism of pyroptosis not previously described in CRC and different from that induced by currently used chemotherapeutic anticancer drugs, which is mediated by activation of caspase-3 and GSDME (Wang et al., 2017; Aizawa et al., 2020). Instead, the nanotoxin activates the inflammasome to activate caspase-1 followed by GSDMD. In this regard, resistance to the classical chemotherapy regimens is an important problem in different types of cancer treatment, including CRC. Exposure to 5-fluorouracil can cause tumor relapse by the emergence of a population of cancer cells with stem-like properties, resistant to this drug (Xu et al., 2015; Francipane et al., 2019). Cancer cells acquire resistance to chemotherapy through the emergence of genetic mutations or epigenetic changes, favoring the activation of signaling pathways related to chemotaxis, cell survival or proliferation, including the CXCL12/CXCR4 axis (Touil et al., 2014). In human cancers, the anti-apoptotic proteins (e.g. Bcl-2, Bcl-XL, and Mcl1) are often upregulated in cancer cells, enabling them to evade apoptotic cell death and losing the capacity to undergo apoptosis in response to chemotherapeutic drugs (Wilson et al., 2006; Pan et al., 2016). The Bcl-2 gene has been shown to be overexpressed in many solid tumor cell lines (Yang et al., 2004; Yip & Reed, 2008; Goldsmith et al., 2012) and clinically, a high Bcl-2 expression in patient samples correlates with a poor response to therapy (Geng et al., 2013). Furthermore, it has been demonstrated that the downregulation of Bcl-2 and Bcl-XL using antisense techniques was able to sensitize cells to chemotherapy (Hayward et al., 2004), whereas a loss of Bax (pro-apoptotic protein) expression resulted in increased resistance (Zhang et al., 2000). Therefore, treatment with the T22-PE24-H6 nanotoxin could represent a promising tool for targeting CXCR4^+^ cancer stem cells and rendering sensitive the CRC tumors with acquired resistance to chemotherapy-induced apoptosis after their treatment, by inducing pyroptosis as an alternative cell death mechanism (Figure 5).

The exotoxin of *Pseudomonas aeruginosa* is being used in the clinical setting in the form of immunotoxins for cancer therapy. Many toxin-based therapies are under clinical trials, but Moxetumomab pasudotox, composed of an anti-CD22 antibody fused to a 38 kDa portion of the *Pseudomonas* exotoxin A, is one of the few approved immunotoxins by the FDA, in this case for the treatment of hairy-cell leukemia (Kreitman et al., 2012). However, immunotoxin therapy has been successfully applied against hematological malignancies, despite of several issues that still represent significant barriers to their effective use for solid cancer treatment (Shan et al., 2013). These hurdles include dose-limiting toxicities, immunogenicity, and reduced efficacy in cytosolic delivery (Kim et al., 2020). We believe that the T22-PE24-H6 nanotoxin, displays a wider therapeutic window than immunotoxins, based on the fact that this nanotoxin is a self-assembled oligomeric construction expectedly formed by around 10–12 protein monomers, that therefore displays 10–12 T22 ligands and that consequently shows super selectivity (Martinez-Veracoechea & Frenkel, 2011; Sharma et al., 2012). Thus, its multivalency makes this construct internalize faster and more intensively in target cancer cells that overexpress the CXCR4 receptor than immunotoxins, which only display two Fab fragments per one exotoxin domain in each immunotoxin molecule, to interact and internalize in target cancer cells. On the other hand, most of the developed fused immunotoxins used the PE38 fragment of the exotoxin. Recent studies demonstrated that de-immunization of recombinant toxins by identifying and removing B-cell epitopes, decreased the immune response when administered in mice. Investigations have focused, for instance, on the elimination of the B-cell epitopes of the 25 kDa portion (HA22-LR-8M) of the PE toxin to produce a fully cytotoxic protein effective against leukemia cell lines (Onda et al., 2011). Therefore, we incorporated the PE24 toxin into our T22-based nanoparticles, which has been demonstrated to be less immunogenic and better, tolerated than previous versions of this toxin, as we observed in this study.

## 5. Conclusion

We have developed an intrinsically cytotoxic and de-immunized nanotoxin that offers a novel therapeutic approach, which reaches a wider therapeutic index, because of its multivalency, than the previously developed nanoconjugates used to treat metastasis in CRC models. Most importantly, we here demonstrate for the first time that the T22-PE24-H6 nanotoxin induces GSDMD-mediated pyroptotic cell death in CRC cells but is unable to trigger apoptosis. Thus, this nanotoxin could be a promising tool for effectively overcoming the apoptosis blockade associated with relapsed chemotherapeutic-resistant and metastatic CRC tumors, by triggering pyroptosis, an alternative cell death pathway, mechanistically different from apoptosis, and poorly studied in oncotherapy.
